# Ipsilateral free semitendinosus tendon graft transfer for reconstruction of chronic tears of the Achilles tendon

**DOI:** 10.1186/1471-2474-9-100

**Published:** 2008-07-08

**Authors:** Nicola Maffulli, Umile Giuseppe Longo, Nikolaos Gougoulias, Vincenzo Denaro

**Affiliations:** 1 Department of Trauma and Orthopaedic Surgery, University Hospital of North Staffordshire, Keele University School of Medicine, Stoke on Trent, ST4 7LN, UK; 2 Department of Orthopaedic and Trauma Surgery, Campus Biomedico University, Via Alvaro del Portillo, 200, 00128 Trigoria, Rome, Italy

## Abstract

**Background:**

Many techniques have been developed for the reconstruction of the Achilles tendon in chronic tears. In presence of a large gap (greater than 6 centimetres), tendon augmentation is required.

**Methods:**

We present our method of minimally invasive semitendinosus reconstruction for the Achilles tendon using one para-midline and one midline incision.

**Results:**

The first incision is a 5 cm longitudinal incision, made 2 cm proximal and just medial to the palpable end of the residual tendon. The second incision is 3 cm long and is also longitudinal but is 2 cm distal and in the midline to the distal end of the tendon rupture. The distal and proximal Achilles tendon stumps are mobilised. After trying to reduce the gap of the ruptured Achilles tendon, if the gap produced is greater than 6 cm despite maximal plantar flexion of the ankle and traction on the Achilles tendon stumps, the ipsilateral semitendinosus tendon is harvested. The semitendinosus tendon is passed through small incisions in the substance of the proximal stump of the Achilles tendon, and it is sutured to the Achilles tendon. It is then passed beneath the intact skin bridge into the distal incision, and passed from medial to lateral through a transverse tenotomy in the distal stump. With the ankle in maximal plantar flexion, the semitendinosus tendon is sutured to the Achilles tendon at each entry and exit point

**Conclusion:**

This minimally invasive technique allows reconstruction of the Achilles tendon using the tendon of semitendinosus preserving skin integrity over the site most prone to wound breakdown, and can be especially used to reconstruct the Achilles tendon in the presence of large gap (greater than 6 centimetres).

## Background

The Achilles tendon (AT) is the most commonly ruptured tendon in the human body [1]. About 20% of complete ruptures of the AT are diagnosed late. The management of chronic ruptures of tendo Achillis is usually different from that of acute rupture, as the tendon ends normally will have retracted [1,2]. The blood supply to this area is relatively poor, and the tendon ends have to be freshened to allow healing. Due to the increased gap, primary repair is not generally possible [2]. Operative procedures for reconstruction of the AT include flap tissue turn down using one [3,4] and two flaps [5], local tendon transfer [6-9], and autologous hamstring tendon harvesting [10]. All of these techniques use a single longitudinal incision for exposure. Following these procedures, complications, especially wound breakdown and infection (9%) [11], are not infrequent, are probably related to the paucity of the soft tissue vascularity, and may require plastic surgical procedures to cover significant soft tissue defects [12].

We have recently described our minimally invasive technique of peroneus brevis reconstruction for the AT using two para-midline incisions [13]. This technique allows reconstruction of the AT using peroneus brevis preserving skin integrity over the site most prone to wound breakdown.

During surgery, sometimes, after trying to reduce the gap of the ruptured AT, the gap produced is greater than 6 cm despite maximal plantar flexion of the ankle and traction on the AT stumps [1]. In such instances, peroneus brevis is not sufficient to full the gap, and ipsilateral hamstring tendon harvesting can be an option [9].

We present our method of minimally invasive semitendinosus reconstruction for the AT. Our technique uses one proximal para-midline incision and one distal midline incision preserving skin integrity over the site most prone to wound breakdown.

## Methods

All procedures described in the present article were approved by the Local Ethics Committee of the Keele University School of Medicine, Stoke on Trent, United Kingdom and all patients gave their written consent.

## Results

### Technical Description

**Figure 1:**
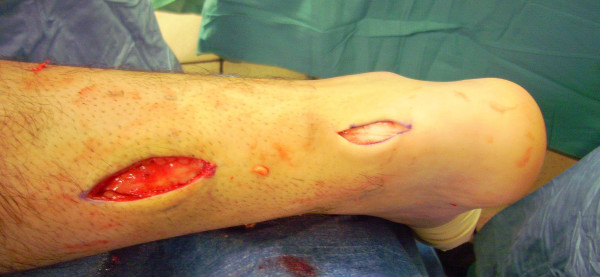
**Two skin incisions are made.** The first incision is a 5 cm longitudinal incision, made 2 cm proximal and just medial to the palpable end of the residual tendon. The second incision is 3 cm long and is also longitudinal but is 2 cm distal and in the midline over the distal end of the tendon rupture.

The patient is positioned prone with a thigh tourniquet. Skin preparation is performed in the usual fashion, and sterile drapes are applied. Pre-operative anatomical markings include the palpable tendon defect and both malleoli. Two skin incisions are made (Figure 1), and accurate haemostasis by ligation of the larger veins and diathermy of the smaller ones is performed. The first incision is a 5 cm longitudinal incision, made 2 cm proximal and just medial to the palpable end of the residual tendon. The second incision is 3 cm long and is also longitudinal but is 2 cm distal and in the midline over the distal end of the tendon rupture. Care is taken to prevent damage to the sural nerve. At the level of the AT insertion, the sural nerve is 18.8 mm lateral to the tendon but, as it progresses proximally, the nerve gradually traverses medially crossing the lateral border of the tendon 9.8 cm proximal to the calcaneum [14]. Thus, the second incision avoids the sural nerve by being placed medial to the nerve.

**Figure 2:**
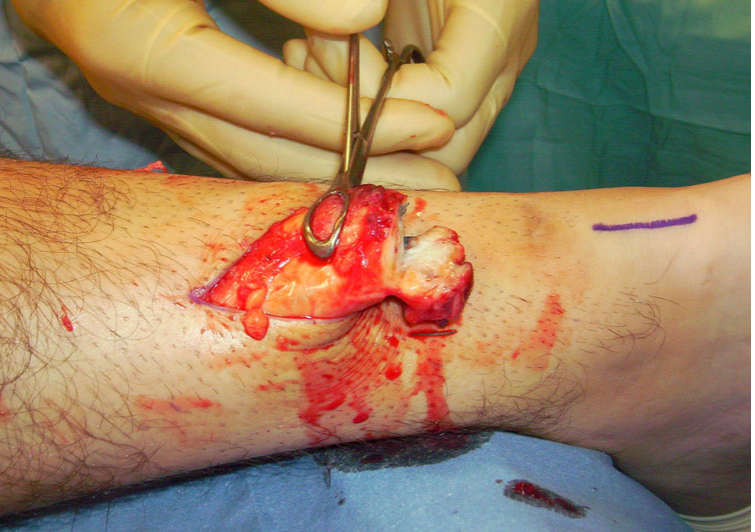
**The proximal and distal AT stump are mobilised, freeing them of all the peritendinous adhesions.** The ruptured tendon end is then resected back to healthy tendon.

**Figure 3:**
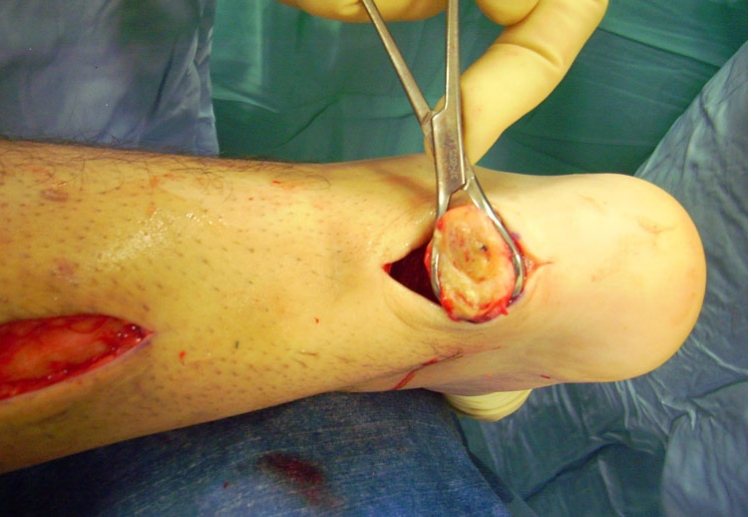
**The proximal and distal AT stump are mobilised, freeing them of all the peritendinous adhesions.** The ruptured tendon end is then resected back to healthy tendon.

**Figure 4:**
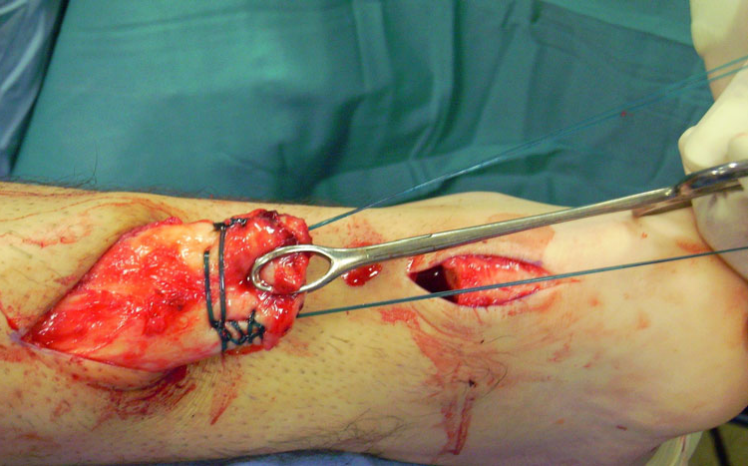
A locking suture is run along the free tendon edge to prevent separation of the bundles.

The proximal and distal AT stump are mobilised, freeing them of all the peritendinous adhesions (Figure 2, 3). It should be possible to palpate the medial tubercle of the calcaneum. The ruptured tendon end is then resected back to healthy tendon, and a Number 1 Vicryl (Ethicon, Edinburgh) locking suture is run along the free tendon edge to prevent separation of the bundles (Figure 4).

The proximal tendon is then mobilised from the proximal wound, any adhesions are divided, and further soft tissue release anterior to the soleus and gastrocnemius allows maximal excursion, minimising the gap between the two tendon stumps. A Vicryl locking suture is run along the free tendon edge to allow adequate exposure and to prevent separation of the bundles.

After trying to reduce the gap of the ruptured AT, if the gap produced is greater than 6 cm despite maximal plantar flexion of the ankle and traction on the AT stumps, the ipsilateral semitendinosus is harvested.

**Figure 5:**
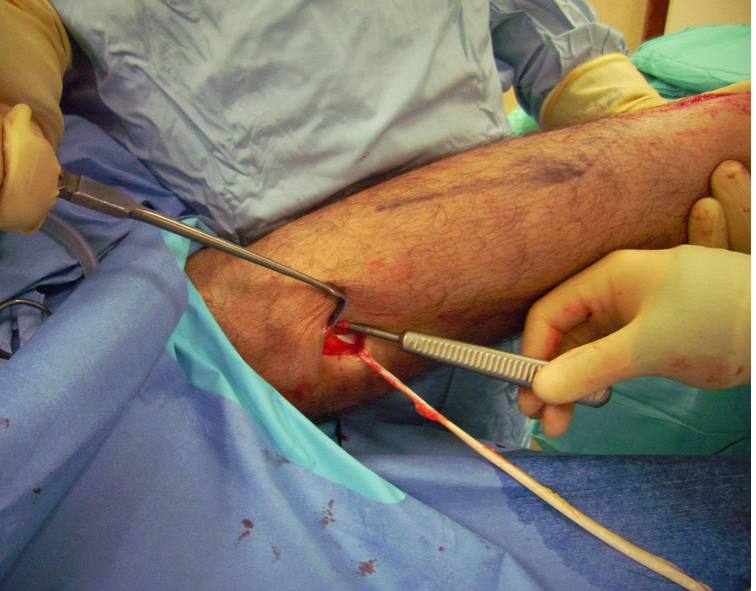
The tendon of the semitendinosus is harvested through a vertical, 2.5–3 cm longitudinal incision over the pes anserinus.

**Figure 6:**
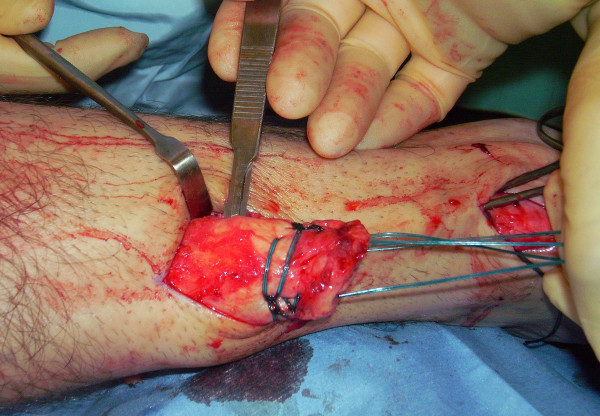
The semitendinosus tendon is passed through a small incision in the substance of the proximal stump of the AT.

**Figure 7:**
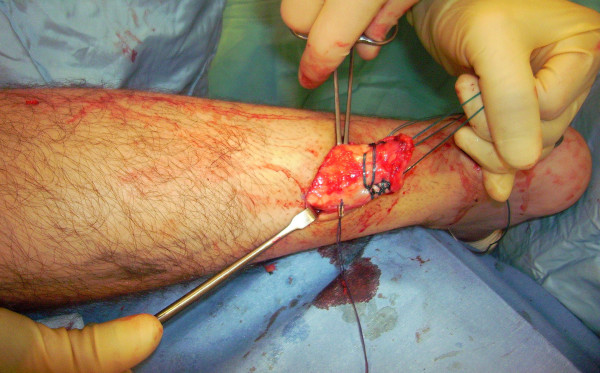
The semitendinosus tendon is passed through a small incision in the substance of the proximal stump of the AT.

**Figure 8:**
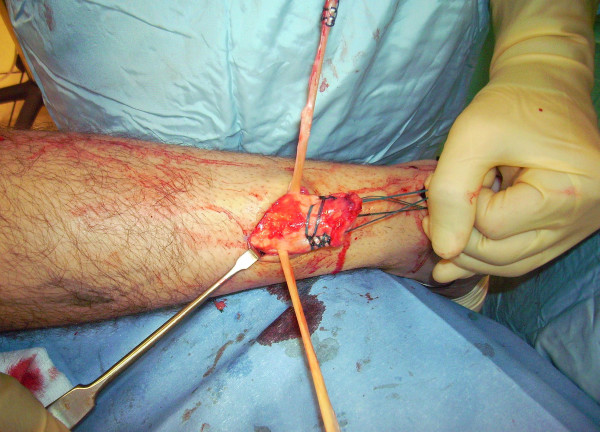
The semitendinosus tendon is passed through a small incision in the substance of the proximal stump of the AT.

**Figure 9:**
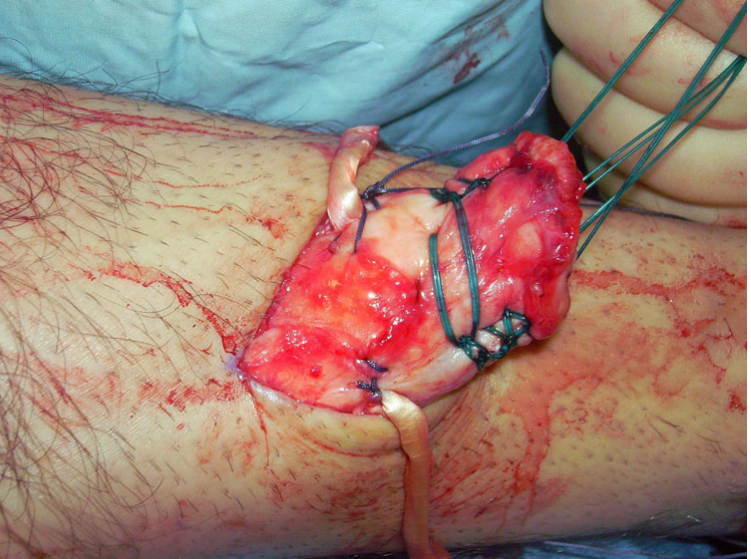
The semitendinosus tendon is sutured to the AT.

**Figure 10:**
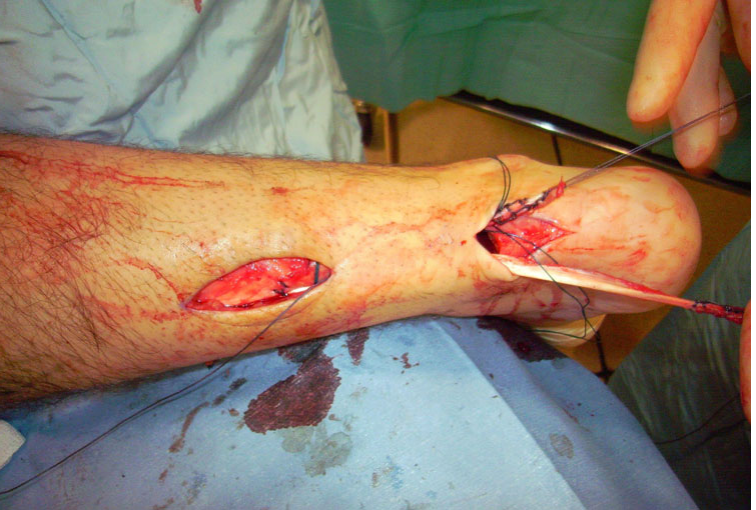
The semitendinosus tendon is then passed beneath the intact skin bridge into the distal incision (Figure 10).

**Figure 11:**
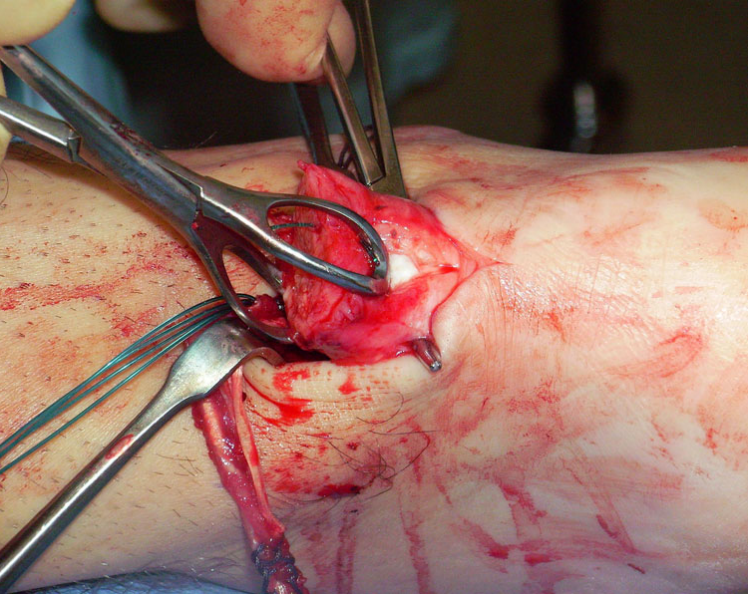
The semitendinosus tendon is passed from medial to lateral through a transverse tenotomy in the distal stump.

**Figure 12:**
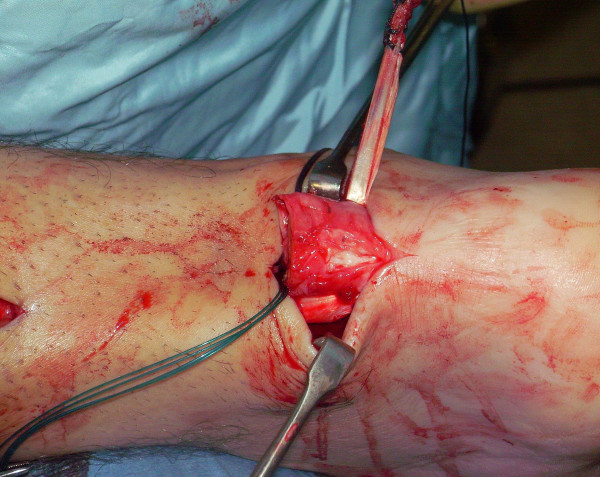
The semitendinosus tendon is passed from medial to lateral through a transverse tenotomy in the distal stump.

**Figure 13:**
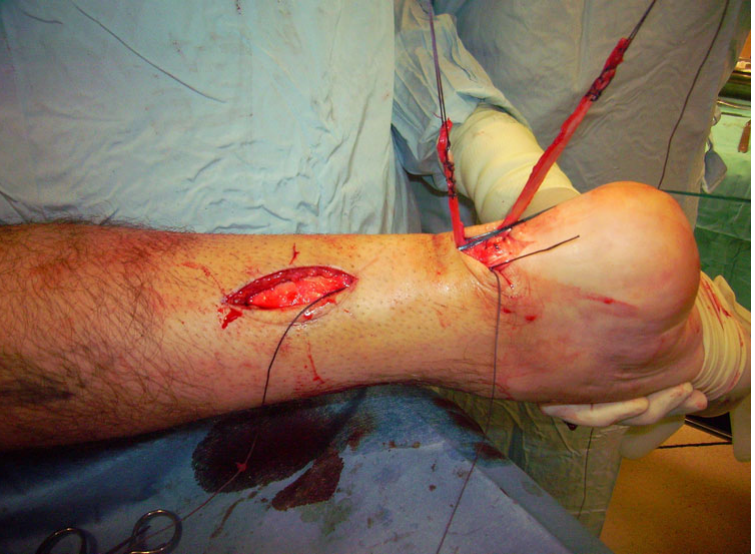
The semitendinosus tendon is passed from medial to lateral through a transverse tenotomy in the distal stump.

The tendon of the semitendinosus is harvested through a vertical, 2.5–3 cm longitudinal incision over the pes anserinus (Figure 5). The semitendinosus tendon is passed through a small incision in the substance of the proximal stump of the AT (Figure 6, 7, 8), and it is sutured to the AT (Figure 9) at the entry and exit point using 3-0 Vicryl (Polyglactin 910 braided absorbable suture; Johnson & Johnson, Brussels, Belgium). The semitendinosus tendon is then passed beneath the intact skin bridge into the distal incision (Figure 10), and passed from medial to lateral through a transverse tenotomy in the distal stump (Figure 11, 12, 13). With the ankle in maximal plantar flexion, the semitendinosus tendon is sutured to the AT at each entry and exit point using 3-0 Vicryl (Polyglactin 910 braided absorbable suture; Johnson & Johnson, Brussels, Belgium). The repair is tensioned to maximal equines.

**Figure 14:**
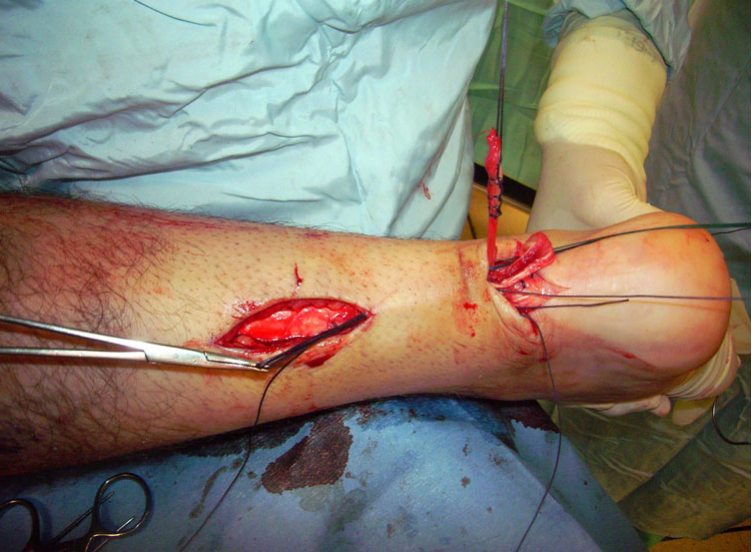
One extremity of the semitendinosus tendon is then passed again beneath the intact skin bridge into the proximal incision.

**Figure 15:**
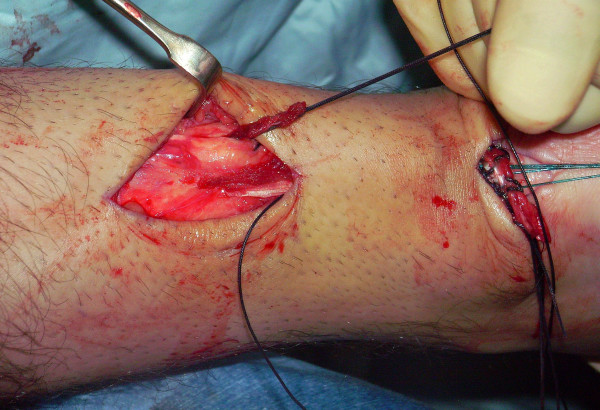
One extremity of the semitendinosus tendon is passed from medial to lateral through a transverse tenotomy in the proximal stump.

**Figure 16:**
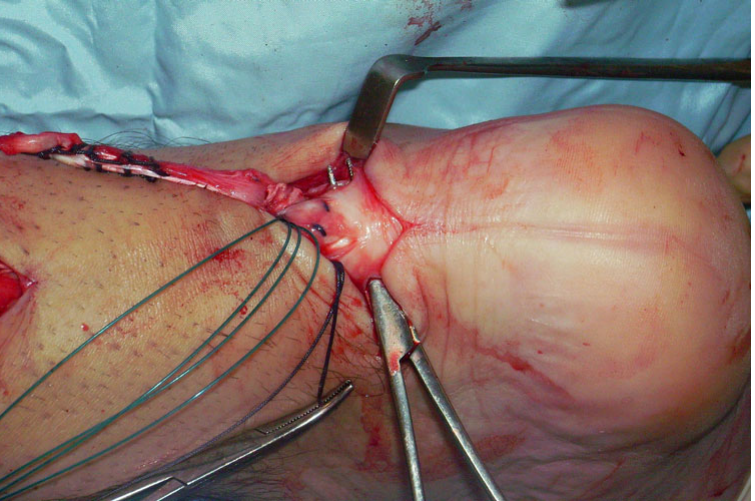
The other extremity of the semitendinosus tendon is then passed again from medial to lateral through a transverse tenotomy in the distal stump.

**Figure 17:**
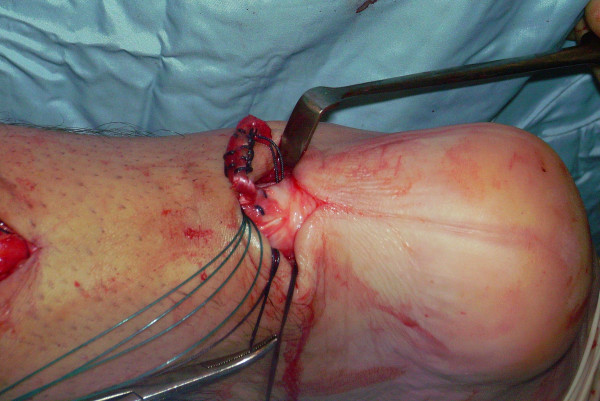
The other extremity of the semitendinosus tendon is then passed again from medial to lateral through a transverse tenotomy in the distal stump.

**Figure 18:**
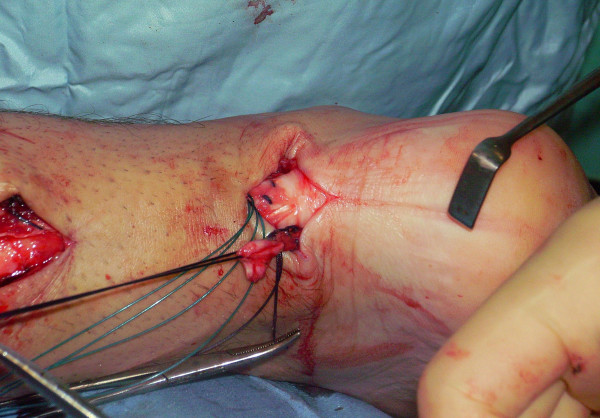
The other extremity of the semitendinosus tendon is then passed again from medial to lateral through a transverse tenotomy in the distal stump.

**Figure 19:**
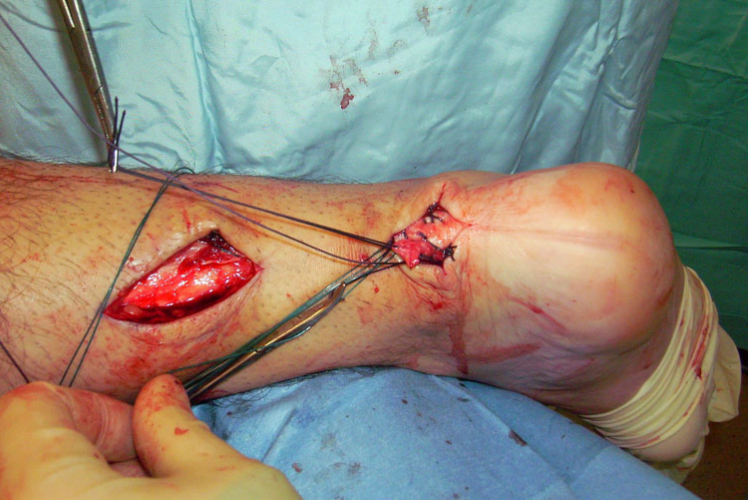
The other extremity of the semitendinosus tendon is then passed again from medial to lateral through a transverse tenotomy in the distal stump.

**Figure 20:**
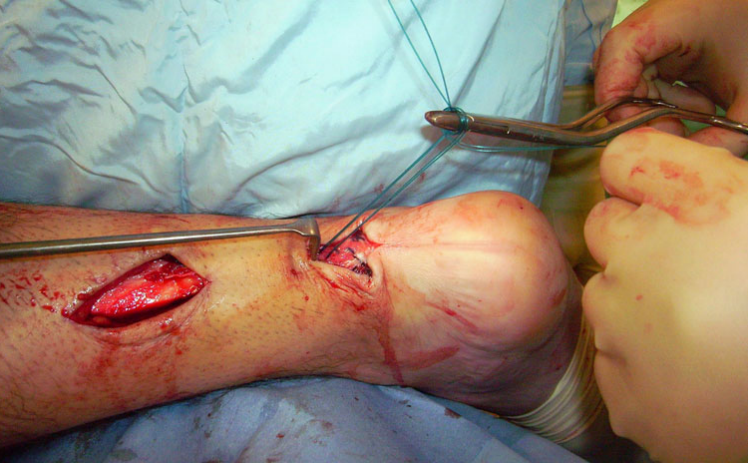
The reconstruction may be further augmented using a Maxon.

**Figure 21:**
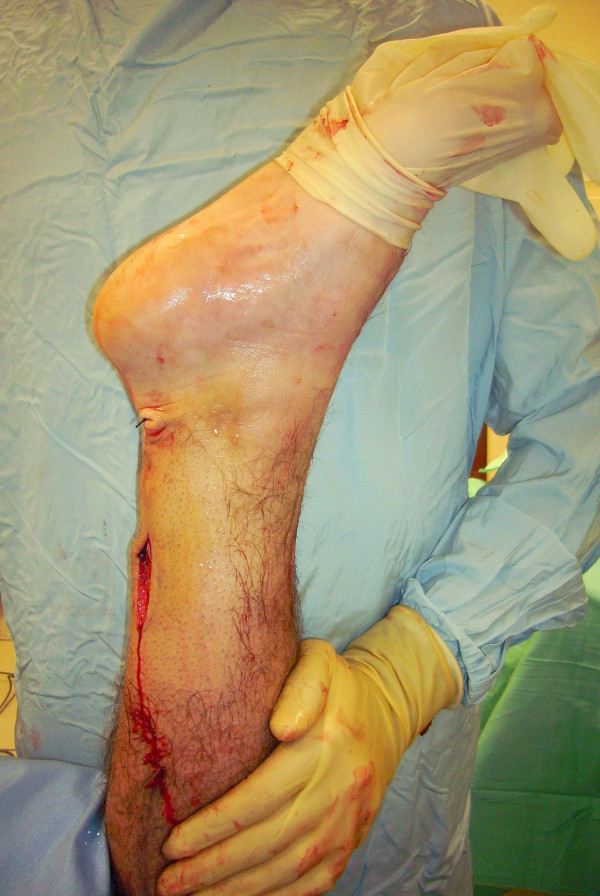
The final result.

**Figure 22:**
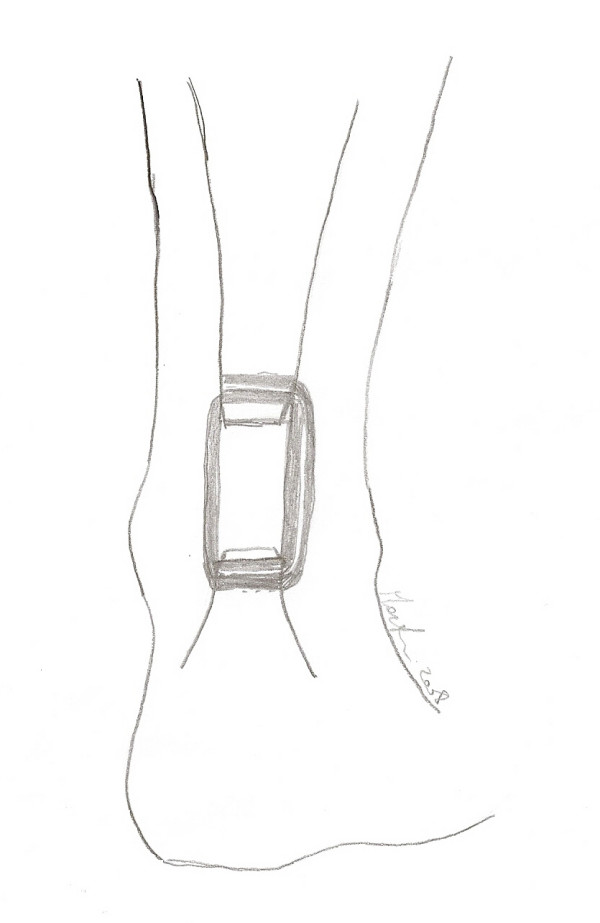
A schematic drawing showing the final result.

One extremity of the semitendinosus tendon is then passed again beneath the intact skin bridge into the proximal incision (Figure 14), and passed from medial to lateral through a transverse tenotomy in the proximal stump (Figure 15). The other extremity of the semitendinosus tendon is then passed again from medial to lateral through a transverse tenotomy in the distal stump (Figure 16, 17, 18, 19). The reconstruction may be further augmented using a Maxon (Tyco Health Care, Norwalk, CT) suture (Figure 20, 21, 22). The wounds are closed with 2.0 Vicryl, 3,0 Biosyn (Tyco Health Care, Norwalk, CT) and Steri-strips (3 M Health Care, St Paul, MN). A previously prepared removable scotch cast support with Velcro straps is applied.

Post operatively, patients are allowed to weight bear as comfort allows with the use of elbow crutches [15,16]. It would be unusual for a patient to weight bear fully at this stage. After 2 weeks, the back shell is removed, and physiotherapy is commenced with the front shell in situ preventing dorsiflexion of the ankle, focusing on proprioception, plantar-flexion of the ankle, inversion and eversion [15,16]. During this period of rehabilitation, the patient is permitted to weight bear as comfort allows with the front shell in situ although full weight bearing rarely occurs on account of balance difficulties and patients usually still require the assistance of a single elbow crutch as this stage. The front shell may be finally removed after 6 weeks. We do not use a heel raise after removal of the cast, and patients normally regain a plantigrade ankle over two or three weeks [15,16].

## Discussion

The principal findings of the present study are that our new method of minimally invasive semitendinosus reconstruction for the AT can be an option to reconstitute the integrity of the flexor apparatus of the foot in patients with a gap greater than 6 cm.

Chronic ruptures of the AT pose a technical challenge, with more complications in patients with a neglected rupture. When compared with open repair in patients with a fresh rupture, reconstruction of the AT in patients with a neglected rupture takes longer, and this may account for some of the wound problems [1,17,18].

Wound breakdown is a challenging complications in AT reconstruction surgery, with open techniques having a 9% superficial infection rate [11]. To minimize the rate of infection after AT reconstruction, we already described a minimally invasive technique of reconstruction of AT using peroneus brevis [13]. That technique allows reconstruction of the AT using peroneus brevis preserving skin integrity, and can be especially used to reconstruct the AT in the presence of previous surgery [13].

However, during surgery it may occurs that, after trying to reduce the gap of the ruptured AT, the gap produced is greater than 6 cm despite maximal plantar flexion of the ankle and traction on the AT stumps [9]. In such instances, peroneus brevis is not sufficient to fill the gap, and tendon augmentation can be required. In neglected ruptures with a large gap, turn down flaps have been advocated. However, in these patients, the quality of the proximal stump from which the turn down flaps are prepared is often suboptimal, and the flaps may still need reinforcement with another tendon. The tendon of semitendinosus is long and strong and provides a robust reconstruction to the AT. We already described the use of a free gracilis tendon graft in this condition [9] and recently semitendinosus tendon augmentation has been described for a large defect after AT rupture in 2 patients [19]. A free semitendinosus graft does not deprive the foot of motor strength and power, is safe, and, given its length, can be used to bridge large gaps. The great advantage of this technique is that it allows to perform a semitendinosus tendon augmentation in a minimally invasive fashion, preserving skin integrity.

To our knowledge, this is the first time that a reconstruction of the AT using a free semitendinosus tendon graft is performed in a minimally invasive fashion. Open reconstruction techniques use relatively long longitudinal incisions, and, in many instances, have incorporated plastic surgical flap procedures to facilitate skin closure [20,21].

Following surgery, the ankle is kept in equinus to prevent disruption of the reconstruction. Vascularity of the soft tissues is maximal at 20° of plantar flexion, and at 40° of plantar flexion the blood supply of the skin is reduced by 49% [22]. Therefore, the tightness of the repair may influence wound healing.

In patients with chronic ruptures, the skin over the gap retracts over several weeks, and remains so until the operation. In open surgery, this skin is incised, and is then stretched out in a relatively acute fashion to accommodate the reconstructed tendon. Therefore, following the reconstruction, the skin over the gap may well be stretched so much that vascular supply is impaired [9]. The reconstructed gastro-soleus AT complex will stretch with increased loading and range of movement exercises during rehabilitation [13].

Preservation of skin cover during reconstruction procedures is clearly an advantage, as the skin is not injured by the operation, and protects the reconstruction beneath. As with all surgery performed through minimally invasive incisions, this procedure is technically demanding. Careful incision placement is required together with skin retraction to allow visualisation of the tendon ends and to permit the reconstruction. This technique is designed to preserve skin cover of the reconstruction site, and, although reconstruction is always risky, it may extend the indications for surgery in patients prone to wound complications such as vasculopaths and diabetics who present with a large gap.

We acknowledge that this is a technical note, and no data about outcome of our patients are presented. However, our preliminary results are good and we plan to evaluate the long term results of this technique. This will be the subject of future endeavours.

## Conclusion

This technique allows minimally invasive reconstruction of the AT using semitendinosus tendon preserving skin integrity, and can be especially used to reconstruct the AT in the presence of large gap (greater than 6 cm).

## Competing interests

The authors declare that they have no competing interests.

## Authors' contributions

NM, UGL, and VD conceived the study. NM, UGL and NG performed the review of the literature. UGL wrote the initial draft. UGL and NG consented the patients whose photos are shown in this manuscript. NM and VD advised on the practicalities of the surgery. All authors read and approved the final manuscript. No funding has been received for the study.

## Pre-publication history

The pre-publication history for this paper can be accessed here:


